# Development of Highly Sensitive Immunosensor for Clenbuterol Detection by Using Poly(3,4-ethylenedioxythiophene)/Graphene Oxide Modified Screen-Printed Carbon Electrode

**DOI:** 10.3390/s18124324

**Published:** 2018-12-07

**Authors:** Nurul Ain A. Talib, Faridah Salam, Yusran Sulaiman

**Affiliations:** 1 Functional Devices Laboratory, Institute of Advanced Technology, Universiti Putra Malaysia, Serdang 43400, Selangor, Malaysia; nurulaina.talib@gmail.com; 2 Department of Chemistry, Faculty of Science, Universiti Putra Malaysia, Serdang 43400, Selangor, Malaysia; 3 Biodiagnostic-Biosensor Programme, Biotechnology and Nanotechnology Research Centre, Malaysian Agricultural Research and Development Institute, Serdang 43400, Selangor, Malaysia; faridahs@mardi.gov.my

**Keywords:** clenbuterol, poly(3,4-ethylenedioxythiophene), graphene oxide, immunosensor, electrochemical

## Abstract

Clenbuterol (CLB) is an antibiotic and illegal growth promoter drug that has a long half-life and easily remains as residue and contaminates the animal-based food product that leads to various health problems. In this work, electrochemical immunosensor based on poly(3,4-ethylenedioxythiophene)/graphene oxide (PEDOT/GO) modified screen-printed carbon electrode (SPCE) for CLB detection was developed for antibiotic monitoring in a food product. The modification of SPCE with PEDOT/GO as a sensor platform was performed through electropolymerization, while the electrochemical assay was accomplished while using direct competitive format in which the free CLB and clenbuterol-horseradish peroxidase (CLB-HRP) in the solution will compete to form binding with the polyclonal anti-clenbuterol antibody (Ab) immobilized onto the modified electrode surface. A linear standard CLB calibration curve with R^2^ = 0.9619 and low limit of detection (0.196 ng mL^−1^) was reported. Analysis of milk samples indicated that this immunosensor was able to detect CLB in real samples and the results that were obtained were comparable with enzyme-linked immunosorbent assays (ELISA).

## 1. Introduction

Clenbuterol (CLB) residue in the meat-based product is a big threat to global food safety. CLB is one of the β-adrenergic stimulating drugs that is able to increase the muscular mass and decrease fat accumulation simultaneously. CLB has a long withdrawal period in animals, thus the residue can easily remain in the meats, blood, milk, and urines [1,2]. Any human that consumes food containing clenbuterol-fed animal may experience health problems such as respiratory problem, increase in heart rate and muscular tremor. Food poisoning due to the consumption of CLB contaminated meat was reported in Italy and clinical symptoms, such as headache, palpitations, distal tremors, and tachipnoea-dyspnoea were diagnosed with 15 people affected [3]. Meanwhile, in Turkey, 68.3% of 41 milk samples were found contaminated with CLB, in which 21.7% were over the acceptable level by the European Union [4]. CLB is still illegally used in the livestock animals, such as swine, cattle, and horse as animal growth enhancer due to economic reason.

In order to ensure the safety of meat-based food products, regular screening, and monitoring of antibiotic residues are necessary. The chromatographic methods such as liquid chromatography-mass spectrometry (LC-MS) [5], high-performance liquid chromatography (HPLC) [6] and gas chromatography-mass spectrometry (GC-MS) [7] are reliable techniques to determine CLB in real samples. The complicated sample preparation steps must be performed by the skilled person to operate the chromatographic instruments, which will easily cause loss or reduce the amount of analyte during the extraction process and it results in inaccurate analysis. Therefore, simple detection methods with less complicated operating procedures may offer an alternative to the above methods for screening and monitoring purposes. Enzyme-linked immunosorbent assays (ELISA) is the common alternative method for rapid CLB screening due to their relatively simple method, cheaper and large samples throughput. At present, ELISA kits for CLB and other antibiotics detection are commercially available [8]. However, an investigation that was performed by Hahnau et al. (1996) to evaluate the performance of these commercial ELISA kits (from eight manufacturers) revealed that not all of these commercial products were reliable and some of them produced interassay precision with more than 10% [9]. Recently, various types of biosensors were developed for a wide range of sensing applications including antibiotics screening and monitoring. These sensors offer high sensitivity and rapid result with a simpler operating procedure to substitute the chromatographic and ELISA methods in the detection of CLB. Detection of CLB based on electrochemical immunosensors was reported previously with low detection limit and a wide range of concentrations [10,11,12]. The electrochemical methods have received more attention due to the high performance in term of sensitivity and selectivity detection for analytical applications [13]. The utilization of the electrochemical sensor in an analytical purpose is widening due to the possibility of instrument miniaturization [14], which is suitable for the on-site application.

Development of electrochemical sensors to the point-of-care testing requires simple, practical and user-friendly setup. In order to comply with these requirements, screen-printed electrodes (SPEs) have emerged as a practical alternative for this purpose [15]. Small and simple strips were developed in various studies [16,17] to offer simple and practical miniaturization electrochemical analysis system that is not only friendly to the users from diverse background and expertise level, but also relatively easy and more cost-effective in terms of manufacturing process [18]. SPEs can be easily modified with various materials such as conducting polymers, metals and nanomaterials via various methods to enhance the sensor performance. This modification also benefitted certain applications, such as the development of biosensors, which usually require a high surface area for the attachment of bio-receptors, such as enzymes, DNAs, antibodies, and cells [19].

Conducting polymers have become essential in both sensor and biosensor designs. Sensors that are based on conducting polymer are able to enhance the effective feature of the electrochemical sensors according to its capability as an electrical carrier. Conducting polymer composites are strongly sensitive to the wide range of analytes, excellent electrical conductivity, easily adjustable to meet sensor requirements, compatible with biological elements compared to metals and ceramics, suitable for miniaturization and mass production of sensors tools [20]. Poly(3,4-ethylenedioxythiophene) (PEDOT) has good stability in the oxidized state and it has excellent thermal stability, thus being suitable for the development of biosensors, since incubation at various temperatures is common in the fabrication of biosensors. Various biological elements are compatible with PEDOT, such as DNAs [21,22], cells [23], and antibodies [24], thus making this polymer suitable for biosensor applications. Polymerization of this polymer can be performed through the electropolymerization method that will produce a highly reproducible polymer film, controllable film thickness, and the polymer is directly obtained in its conducting state [25].

In order to improve the electrochemical properties of conducting polymers, the incorporation of these polymers with carbon materials can be performed. Conducting polymer composites with carbon materials, such as graphene oxide (GO), can be prepared due to the ability of GO that can easily disperse in the aqueous solutions. GO can form a composite with conducting polymers, such as polypyrrole/graphene oxide (PPy/GO), poly(3,4-ethylenedioxythiopehene/graphene oxide (PEDOT/GO), and polyaniline/graphene oxide (PANI/GO) [26]. GO is known for their excellent thermal stability, mechanical and electrical properties [27]. A high surface area [28], ultrafast electron transport [29] and suitability for large-scale manufacturing of GO [30] are very convenient features for sensor development. The presence of abundant carboxyl groups in GO structure is very useful for incorporation of biological molecules by using crosslinker agents, such as 1-ethyl-3-(-3-dimethylaminopropyl) carbodiimide hydrochloride/N-hydroxysulfosuccinimide (EDC/NHSS) to form binding between these molecules [31].

In this work, an immunosensor based on PEDOT/GO modified screen-printed electrode (SPCE) for detection of CLB was developed. Modification of electrode with PEDOT/GO was performed electrochemically and its effect on the sensing performance was evaluated. Detection of CLB was conducted based on direct competitive immunoassay, and the signal produced was determined electrochemically. Application of this immunosensor in real samples was evaluated from spiked milk samples and compared with ELISA.

## 2. Materials and Methods

### 2.1. Materials

3,4-Ethylenedioxythiopehene (EDOT), clenbuterol hydrochloride (CLB), salbutamol, terbutaline hemisulfate salt (terbutaline), nitrofurantoin (nitrofuran), vancomycin hydrochloride (vancomycin), tetracycline, chloramphenicol, streptomycin sulfate salt (streptomycin), di-sodium hydrogen phosphate (Na_2_HPO_4_), sodium dihydrogen phosphate (NaH_2_PO_4_), 4-nitrophenyl phosphate disodium salt hexahydrate (*p*-NPP), *N*-(3-dimethylaminopropyl)-*N*′-ethylcarbodiimide hydrochloride (EDC), N-hydroxysulfosuccinimide (NHSS) and 3,3’,5,5’–tetramethylbenzidine (TMB) were obtained from Sigma Aldrich. Graphene oxide (GO) was purchased from Graphenea. Mabuterol hydrochloride (mabuterol) and ractopamine hydrochloride (ractopamine) were purchased from Fluka. Bicinchoninic acid (BCA) protein assay reagent A (contains sodium carbonate, sodium bicarbonate, BCA detection reagent and sodium tartrate in 0.1 N sodium hydroxide) and BCA protein assay reagent B (containing copper (II) sulfate pentahydrate) were obtained from Thermo Scientific. Clenbuterol-horseradish peroxidase (CLB-HRP) was purchased from Fitzgerald (North Acton, MA, USA). Clenbuterol ovalbumin (CLB-OVA) was purchased from Glory Science (China). Screen-printed carbon electrode (SPCE, DRP C110) was purchased from Dropsens. Polyclonal anti-clenbuterol antibody (Ab) was obtained from a rabbit immunized with clenbuterol bovine serum albumin (CLB-BSA), following the procedures described in the literature [32]. The antibody production protocol was reviewed and approved by the Animal Ethics Committee of Malaysian Agricultural Research and Development Institute, Malaysia (Approval number 20171103/R/MAEC26; Approval date 3 November 2017).

### 2.2. Buffers and Solutions

Phosphate buffer solution (PBS) was prepared by mixing 0.01 M Na_2_HPO_4_ and 0.01 M NaH_2_PO_4_ in deionized water and adjusted to the desired pH accordingly. Crosslinker was prepared by mixing EDC and NHSS in a ratio of 1:1 in 0.01 M PBS pH 7.4. Clenbuterol-horseradish peroxidase (CLB-HRP) was diluted in 0.01 M PBS with a ratio of 1:640, as recommended by the manufacturer (Fitzgerald). The washing buffer was prepared by mixing 0.05% Tween 20 (Sigma Aldrich, St. Louis, MO, USA) with 0.01 M PBS pH 7.4. Dry milk (Blotto, non-fat Santa Cruz Biotechnology, Dallas, TX, USA) was used as a blocking agent by diluting in 0.01 M PBS pH 7.4. All of the solutions were prepared using deionized water (18.2 MΩ cm) from the Milli-Q system (Millipore, Boston, MA, USA).

### 2.3. Preparation of Clenbuterol Immunosensor

#### 2.3.1. Preparation of Poly(3,4-ethylenedioxythiophene)/graphene Oxide Modified Screen-Printed Carbon Electrode

Prior to the surface modification, the SPCE was undergoing pre-treatment by cyclic voltammetry (CV) at −1.5 to 0 V for 3 cycles in 1.0 mM H_2_SO_4_ solution. A mixture containing 0.01 M EDOT and 0.01 mg mL^−1^ GO was drop casted onto SPCE covering the reference electrode (RE), counter electrode (CE), and working electrode (WE) area. The electropolymerization potential was carried out at a constant 1.2 V for 321.84 s using Autolab PGSTATM101 [33].

#### 2.3.2. Preparation of Clenbuterol Immunosensor

Activation of carboxyl functional groups from SPCE/PEDOT/GO surface was performed by adding 10 µL of EDC/NHSS linker solution on the WE surface followed by incubation at ambient temperature for 15 min. The unbound linker was removed through the washing process by rinsing with washing buffer. The electrode was further incubated with 10 µL of Ab solution for 1 h at 46 °C [34], followed by rinsing with washing buffer, resulting SPCE/PEDOT/GO/Ab. The optimum immunoassay conditions i.e., antibody concentration, pH, % blocking, immobilization temperature, and immobilization time have been optimized in our previous study [34].

Blocking step was performed with dry milk as the blocking agent. 20 µL of 0.03% dry milk was incubated on the modified surface at 46 °C for 30 min, followed by rinsing with washing buffer. The direct competitive immunoassay was applied for detection of CLB by incubating 10 µL of standard CLB (5 to 150 ng mL^−1^) and the equal amount of CLB-HRP at 46 °C for 33 min [34]. The unbound CLB was removed by rinsing with washing buffer. Current signal from chronoamperogram was recorded after 50 µL of TMB dropped on the surface. The TMB act as the substrate for HRP, thus the reduction of this TMB that is catalyzed by HRP will produce amperometric respond and the electrochemical signal was recorded for 300 s. The standard calibration graph was plotted and fitted with a linear regression equation, while the limit of detection (LOD) was determined based on Equation (1) [35,36];

(1) $$\mathrm{LOD}={\left(x\frac{a-d}{\left(a-d\right)-3\mathrm{SD}}-1\right)}^{-1/k}$$

where,

SD = standard deviation of zero value;
a = maximum values of calibration curve;
d = minimum values of calibration curve;
*x* = concentration of the EC_50_ value; and,
k = curve hill’s slope.

### 2.4. Characterization

The electrochemical behavior of the developed immunosensor was evaluated based on the CV in the potential range of 0.2 V to 0.6 V (1 mM K_3_[Fe(CN)_6_] mixed with 0.1 M KCl) and electrochemical impedance spectroscopy (EIS) within frequency range from 1 Hz to 10 MHz using sinusoidal current of 5 mV amplitude at open circuit potential (in a solution containing 5 mM K_3_[Fe(CN)_6_], 5 mM K_4_[Fe(CN)_6_], and 0.1 M KCl). All electrochemical measurements were performed in Faraday cage at ambient temperature. The surface morphology of the modified electrode (pre-coated with platinum) was analyzed by using field emission scanning electron microscope (FESEM, JEOL JSM-7600F) and the Ab immobilization was confirmed by BCA protein assay [37]. The BCA protein assay was performed on the electrode at each fabrication stage by adding 100 µL substrate reagent A and reagent B (9:1), followed by incubation at 37 °C for 30 min. The color changes on the electrode surface were observed using ELISA reader at 560 nm absorbance.

### 2.5. Determination of Potential Applied

The optimum potential applied was determined by applying a constant potential (between −0.6 and 0.6 V) for 100 s for standard CLB detection (0 to 250 ng mL^−1^). The preparation procedure is similar as described in Section 2.3.2.

### 2.6. Optimization of Antibody Concentration

Suitable Ab concentration was determined from indirect ELISA to determine antibody titer. Each microplate was filled with 100 µL CLB-OVA (100 µg mL^−1^), followed by overnight incubation at 4 °C. After incubation, the microplate was washed with washing buffer. 250 µL of dry milk (0.05%) was added to each well and was incubated for 1 h at 37 °C, followed by washing with the washing buffer. 100 µL of Ab at various concentrations (10^0^ to 10^−7^ mg mL^−1^) was inserted into each well in three replicates. After being incubated for 2 h at 37 °C, the wells were washed with washing buffer. 4-nitrophenyl phosphate disodium salt hexahydrate (*p*-NPP) was added to each well (100 µL/well), followed by absorbance reading at 405 nm through ELISA reader.

### 2.7. Preparation of Real Samples

Analysis of CLB in real samples was determined from two full cream milk (labeled as milk A and milk B) purchased from local market. Generally, the milk samples were diluted with a ratio of 1:1 with 0.01 M PBS (pH 7.4) [38], followed by vortexing for 1 min. The spiked milk samples were prepared by adding the appropriate concentration of standard CLB diluted in 0.01 M PBS, followed by vortexing for 1 min. Detection of CLB in a milk sample was performed following the procedure described in Section 2.3.2. The analysis result was compared with a direct ELISA method, as described by the procedures in the literature [32,39,40].

## 3. Results and Discussion

### 3.1. The Principle of the Immunosensor

**Figure 1:**
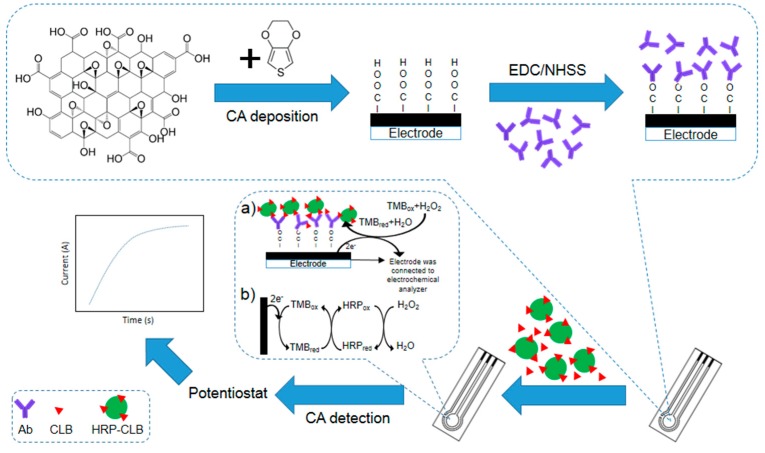
Schematic diagram of fabrication of clenbuterol hydrochloride (CLB) immunosensor. (**a**) Electrochemical immunosensor format used for CLB detection; (**b**) Indirect electron transfer for TMB redox shown by complex TMB/HRP/H_2_O_2_ enzyme reaction on modified SPCE for reduction current formation.

The immunosensor developed for CLB detection in this study is illustrated in Figure 1. The modified SPCE contains abundant carboxyl groups from the GO that utilize to form binding with Ab through EDC/NHSS linker. The Ab acts as a bio-recognition element in this biosensor to ensure high selective performance. GO has a high surface active area, thus allowing more Ab to bind onto the modified electrode surface and increase the sensitivity of the immunosensor. During the redox reaction, background noise from unbound protein may contribute to the false signal. Therefore, dry milk is introduced as a blocking agent to cover the unoccupied electrode surface. A washing step is also applied after each incubation step to remove the unbound protein.

The electrochemical assay applied here is inspired by ELISA. CLB is a small molecule since this antigen is a hapten, thus it provides a little contribution to the electron transfer. In order to increase the signal, direct competitive assay (Figure 1a) is applied. HRP is a common label enzyme used in the immunoassay. The redox activity of this enzyme can be determined by the electrochemical method. During the incubation time, CLB-HRP will compete with free CLB to form binding with Ab immobilized on the electrode surface. As the substrate TMB is added and potential is applied, the reduction of TMB that is catalyzed by HRP (Figure 1b) can be measured. The signal produced is based on the amount of CLB-HRP that is bound onto Ab. If more free CLB in the solution is able to bind with Ab, fewer CLB-HRP will be able to bind into the Ab, thus a smaller current will be recorded and vice versa.

### 3.2. Characterization

#### 3.2.1. Cyclic Voltammetry

**Figure 2:**
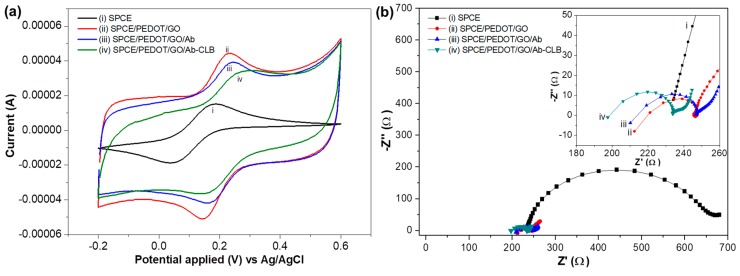
(**a**) Cyclic voltammetrys (CVs) of (i) bare screen-printed carbon electrode (SPCE); (ii) screen-printed carbon electrode poly(3,4-ethylenedioxythiophene)/graphene oxide (SPCE/ PEDOT/GO); (iii) SPCE/PEDOT/GO/Ab; (iv) SPCE/PEDOT/GO/Ab-CLB in 1mM K_3_[Fe(CN)_6_] and 0.1 M KCl; (**b**) electrochemical impedance spectroscopy (EIS) of (i) bare SPCE; (ii) SPCE/PEDOT/GO; (iii) SPCE/PEDOT/GO/Ab; (iv) SPCE/PEDOT/GO/Ab-CLB in 5 mM K_3_[Fe(CN)_6_], 5 mM K_4_[Fe(CN)_6_], and 0.1 M KCl.

Cyclic voltammograms in 1 mM K_3_[Fe(CN)_6_] containing 0.1 M KCl for each modification stage of the electrode are shown in Figure 2a. An increase in peak current is obtained for SPCE/PEDOT/GO (*I*_pa_ = 18.9 µA, *I*_pc_ = −22.9 µA) in comparison with bare SPCE (*I*_pa_ = 18.4 µA, *I*_pc_ = 14.9 µA) due to the high conductivity of PEDOT/GO composites thus improve the electron transfer on the modified electrode [41]. Since GO has a high surface area and consists of abundant of the carboxyl groups, a lot of Ab are able to bind with the carboxyl groups through EDC/NHSS crosslinker and occupied the SPCE surface, thus allowing the occurrence of bioactivity [42]. Ab is a non-conductive material, hence it causes a decrease in the peak current (*I*_pa_ = 14.7 µA, *I*_pc_ = −14.7 µA) of SPCE/PEDOT/GO/Ab. The immobilized Ab is able to capture and form binding with the free CLB and labeled CLB (CLB-HRP). Both Ab and CLB are non-conducting materials, thus as these materials bound to the SPCE surface, SPCE/PEDOT/GO/Ab-CLB becomes less conductive and the peak current of the modified electrode is decreased (*I*_pa_ = 11.9 µA, *I*_pc_ = −14.3).

#### 3.2.2. Electrochemical Impedance Spectroscopy

The modified electrode was further characterized using EIS to study the electrical behavior. The Nyquist plot of bare SPCE in Figure 2b shows a typical impedance spectrum containing a semicircle region at a high frequency that was attributed to the resistance charge transfer (*R*_ct_) and a linear region representing a diffusion process at low frequency region. The SPCE exhibits a relatively large semicircle diameter (*R*_ct_ = 423 Ω), indicating high impedance. However, the impedance decreases (*R*_ct_ = 24 Ω) after PEDOT/GO is deposited on the SPCE due to the increase of conductivity thus improve the electron transfer between the solution and the modified electrode surface [13]. After the immobilization of Ab, the impedance of immunosensor has increased (*R*_ct_ of 33 Ω), indicating the immobilization of Ab on the electrode interface [43]. The non-conductive of Ab has resulted in the difficulty of electron movement, hence, increasing the resistance. After the detection, the additional barrier is formed, implying the capture of CLB by its Ab thus preventing the electron transfer to the modified electrode surface and resulting only slightly increase in impedance (*R*_ct_ = 34 Ω) which confirmed the immobilization of Ab and CLB. However, since CLB is a hapten (very small molecule), only a small increment in impedance and almost negligible resistance occurs, hence do not affect much effect on the *R*_ct_. 

#### 3.2.3. Morphology

**Figure 3:**
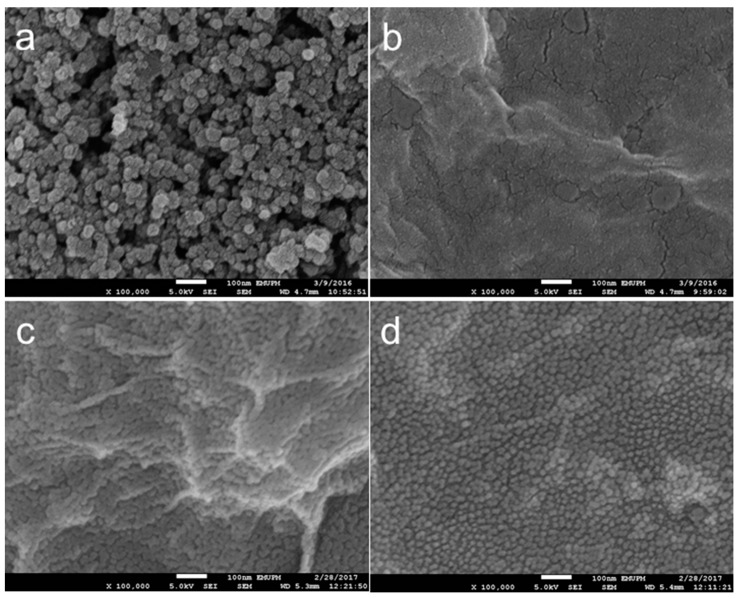
Field emission scanning electron microscope (FESEM) images of (**a**) bare SPCE; (**b**) SPCE/PEDOT/GO; (**c**) SPCE/PEDOT/GO/Ab; and, (**d**) SPCE/PEDOT/GO/Ab-CLB.

Different morphologies for each modification stage are observed from FESEM analysis (100 k magnification). Deposition of PEDOT/GO composites on SPCE has caused the rough surface of bare SPCE (Figure 3a) to turn into a smoother structure with a wrinkled paper-like sheet (Figure 3b). Immobilization of Ab on the electrode has further modified the SPCE/PEDOT/GO into SPCE/PEDOT/GO/Ab. The appearance of a small granular structure on the wrinkle surface (Figure 3c) indicates that Ab has been successfully bound onto the electrode surface. No significant change in the electrode morphology is observed with further immobilization with CLB (SPCE/PEDOT/GO/Ab-CLB) (Figure 3d). Confirmation of Ab binding on the electrode surface is further evaluated by performing BCA protein assay. The existence of protein will cause the green color of mix reagent turns into purple. Ab is a protein, thus the incubation of electrode with mixed reagent has caused the color of the solution to become purple. The binding of Ab on the modified surface has caused the reduction of copper (Cu^2+^) to cuprous (Cu^+^), followed by the chelation with BCA and lead to the formation of BCA/copper complex. Therefore, Ab immobilization on the electrode surface is confirmed. The test was also performed on the bare SPCE and SPCE/PEDOT/GO/ surface, and no changes in the green color of mix reagent are observed.

### 3.3. Determination of Potential Applied

**Figure 4:**
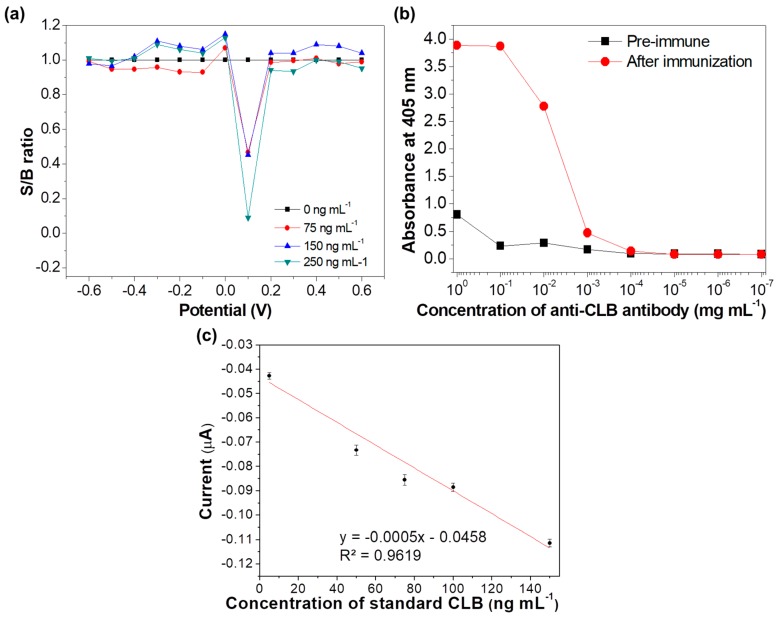
(**a**) Plot signal to background (S/B) for each step potential from −0.6 to 0.6 V with chronoamperometry measurement at various concentrations (0, 75, 150 and 250 ng mL^−1^); (**b**) Enzyme-linked immunosorbent assays (ELISA) titer of Ab activity at various concentrations from 10^0^ to 10^−7^ mg mL^−1^ (the black points represent Ab titer before immunization, while the red points represent Ab titer after immunization); (**c**) Standard immunosensor CLB calibration curve.

The applied potential for CLB determination was determined using step amperometry from −0.6 to 0.6 V for different CLB concentration (0 to 250 ng mL^−1^). The ratio of signal current over background (S/B) for each potential applied and concentration was calculated and displayed in Figure 4a. The pattern of the current signals was evaluated to determine the suitable potential (V) to be applied in this study, since the detection is performed using the CA technique. The potential of 0.1 V shows a larger S/B ratio as compared to the other applied potentials, implying that this potential is the most suitable potential to be applied for current measurement for this immunosensor.

### 3.4. Optimization of Antibody Concentration

The optimum Ab concentration was determined based on the indirect ELISA titer and the highest Ab titer was determined by the lowest concentration of the Ab recognizing a specific antigen (CLB) [44]. Titers of pre-immune bleed and after immunization bleed were analyzed. The absorbance value of Ab after immunization decreases as the Ab concentration decreases from 100 mg mL^−1^ to lower concentrations as shown in Figure 4b, while the Ab titer value was determined as 1:10,000 based on the dilution point where the two absorbances (pre-immune Ab titer and Ab titer after immunization) overlapped. Ab concentration of 10^−1^ and 100 mg mL^−1^ (0.1 and 1 mg mL^−1^) have resulted in the highest absorbance. However, the absorbance value of the Ab after immunization at concentration 0.1 mg mL^−1^ is almost 17-fold higher than the pre-immune in comparison to Ab concentration 1 mg mL^−1^ (five-fold), hence 0.1 mg mL^−1^ was chosen as the optimum Ab concentration for the immunosensor development. The direct competitive electrochemical assay was performed and the standard CLB calibration graph was plotted (Figure 4c). A linear graph is obtained with R^2^ of 0.9619, indicating that the concentration of 0.1 mg mL^−1^ Ab is suitable for this study.

### 3.5. Analytical Performance of the Immunosensor

**Figure 5:**
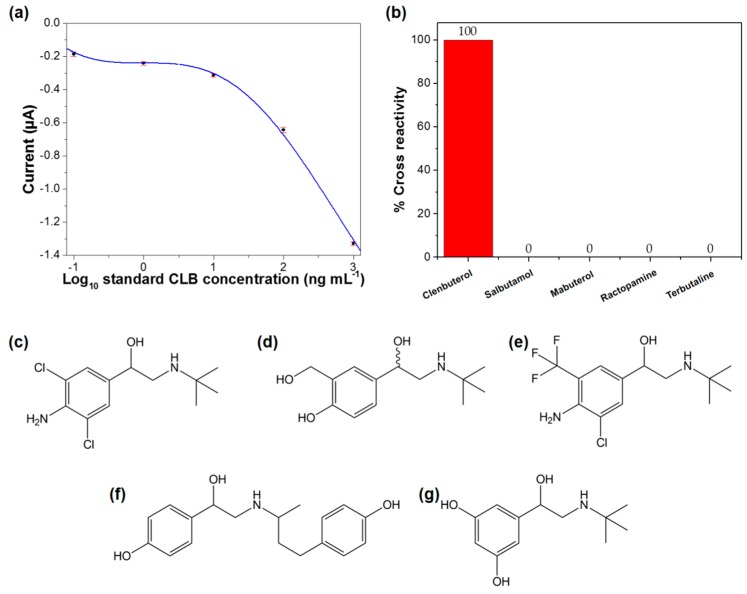
(**a**) The measured currents are fitted to a sigmoidal curve for estimation of limit of detection (LOD) (solid line); (**b**) Immunosensor selectivity against other antibiotics from β-agonist family. Structures of some representative of β–agonist families; (**c**) clenbuterol; (**d**) salbutamol; (**e**) mabuterol; (**f**) ractopamine; and (**g**) terbutaline.

Determination of LOD was performed based on the sigmoidal plot (Figure 5a) and Equation (1). The LOD was estimated as 0.196 ng mL^−1^, which is complied with the requirement of Codex Alimentarius Commission (CAC) regulations (10 ng mL^−1^) [45]. In addition, the obtained LOD is much lower when compared to other reported literature (Table 1). The low LOD reported in this study indicates that this proposed method has a high potential to be used to detect CLB. The performance of the developed immunosensor is comparable with other previously reported methods for CLB detection, as shown in Table 1. The modification of electrode with PEDOT/GO composites has enhanced the electrochemical properties of this immunosensor, hence increase the sensitivity and sensing performance [46]. Thus, fast biosensing tool (detection time is only 5 min (300 s)) was reported in this study. In order to evaluate the reproducibility of this immunosensor, the electrochemical assay was reproduced under the same conditions. The relative standard deviation (RSD) of this reproducibility test is 0.65 (*n* = 7), indicating excellent reproducibility. The storage stability performance of this immunosensor was also evaluated. After storage at 4 °C for a month, 114% of the initial current response was obtained, concluding good storage stability. The analytical performance of this immunosensor was further evaluated based on the selectivity performance. The immunosensor was tested with other antibiotics from β–agonist family i.e., salbutamol, mabuterol, ractopamine, and terbutaline. No cross-reactivity was observed for all these antibiotics, indicating the excellent selectivity of this immunosensor towards CLB detection (Figure 5b). Even though the other tested antibiotics have the almost similar basic structure (Figure 5c–g), analysis of these antibiotics do not produce a significant signal or interfere during the measurements. The polyclonal Ab used as a bio-recognition element in this study is specific against CLB, hence this immunosensor is only selective towards CLB.

### 3.6. Real Samples Analysis

The validation of the developed immunosensor for the detection of CLB in real samples was evaluated based on the analysis of spiked milk samples. The analysis of CLB spiked samples was accomplished by interpolating the measured current values into the calibration plot. The results of the analysis were compared with ELISA analysis, as shown in Table 2. The recoveries of spiked samples are detected in the range of 89.2 to 107.6%, indicating significant reliability for the detection of CLB in real samples using this immunosensor. In comparison to ELISA, comparable results were produced, implying the reliability of this immunosensor for CLB monitoring, and screening in real samples analysis.

## 4. Conclusions

A highly sensitive electrochemical immunosensor for CLB detection was successfully developed. The modification of SPCE with PEDOT/GO as the sensor platform and implementation of direct competitive immunoassay format has successfully utilized for CLB detection. The morphology study revealed uniform immobilization of Ab onto the PEDOT/GO, indicating the suitability of this sensor platform to be developed as a biosensor. The low LOD (0.196 ng mL^−1^) that was reported using this immunosensor is complying with the requirement of CAC regulations. The detection of CLB in spiked milk samples has resulted in 89% and 107% recoveries, thus the developed immunosensor is reliable for real samples analysis.

## Figures and Tables

**Table 1:** Comparison of the analytical performances of developed immunosensor with previous reports method for determination of CLB.

Techniques	Detection Limit	Linear Range	Reference
GC-MS	2 ng mL^−1^	0.06 to 8.0 ng mL^−1^	[47]
Surface-enhanced Raman spectroscopy (SERS)	0.5 ng mL^−1^	0.5 to 20 ng mL^−1^	[48]
Surface-enhanced Raman spectroscopy (SERS)	NA	1 to 1000 pg mL^−1^	[49]
Surface plasmon resonance	1.26 ng mL^−1^	NA	[50]
Quartz crystal microbalance sensor	3.0 ng mL^−1^	NA	[45]
Fluorometry/FRET	3.96 ng mL^−1^	200 to 1800 ng mL^−1^	[51]
Electrochemical sensor	0.64 ng mL^−1^	1.0 to 26.0 ng mL^−1^	[52]
Electrochemical sensor	0.076 ng mL^−1^	0.3 to 100 ng mL^−1^	[53]
Electrochemiluminescence sensor	0.8 ng mL^−1^	5 to 100 ng mL^−1^	[54]
Electrochemical sensor	1.92 ng mL^−1^	10 ng mL^−1^ to 2 μg mL^−1^	[55]
Electrochemical immunosensor	0.196 ng mL^−1^	5 to 150 ng mL^−1^	This work

NA = not available.

**Table 2:** Analysis of milk samples (*n* = 3).

		Immunosensor		ELISA	
Samples	Spiked (ng mL^−1^)	Average Recovery (ng mL^−1^)	Percentage Recovery (%)	Average Recovery (ng mL^−1^)	Percentage Recovery (%)
Milk A	50	44.6 ± 3.23	89.2	69.1 ± 0.59	138
Milk B	50	53.8 ± 9.71	107.6	57.6 ± 0.22	115
